# Expediting multiple biological properties of main bioactive compounds of *Mentha pulegium* L

**DOI:** 10.1186/s13568-025-01911-8

**Published:** 2025-07-14

**Authors:** Nasreddine El Omari, Oumayma Aguerd, Abdelaali Balahbib, Naoual El Menyiy, Mohammed Amanullah, Jactty Chew, Long Chiau Ming, Abdelhakim Bouyahya

**Affiliations:** 1 High Institute of Nursing Professions and Health Techniques of Tetouan, Tetouan, Morocco; 2 Laboratory of Human Pathologies Biology, Department of Biology, Faculty of Sciences, Mohammed V University in Rabat, 10106 Rabat, Morocco; 3 High Institute of Nursing Professions and Health Techniques of Errachidia, Errachidia, Morocco; 4 Laboratory of Pharmacology, National Agency of Medicinal and Aromatic Plants, 34025 Taounate, Morocco; 5 Department of Clinical Biochemistry, College of Medicine, King Khalid University, Abha, Kingdom of Saudi Arabia; 6 Sir Jeffrey Cheah Sunway Medical School, Faculty of Medical and Life Sciences, Sunway University, Sunway City, Malaysia; 7 Datta Meghe College of Pharmacy, Datta Meghe Institute of Higher Education and Research (Deemed to Be University), Sawangi, Wardha, India; 8 School of Pharmacy, Faculty of Medical and Life Sciences, Sunway University, Sunway City, Malaysia

**Keywords:** Human and disease, Diabetes, Infectious disease, Essential oil, Menthone, Pulegone, Dermatoprotective effect

## Abstract

This study aimed to evaluate the antidiabetic, dermatoprotective, and antibacterial properties of *Mentha pulegium* L. essential oil (MPEO), harvested in Ouezzane, northwest Morocco. Gas chromatography-mass spectrometry (GC–MS) analysis identified pulegone (41.0%) and menthone (21.2%) as the major constituents. The antibacterial activity was assessed using the micro-broth dilution method, while antidiabetic and dermatoprotective effects were evaluated via in vitro inhibition of α-amylase, α-glucosidase, tyrosinase, and elastase. Additionally, molecular docking was used to assess interactions between key enzymes and major compounds. Both menthone and pulegone exhibited strong antibacterial effects against *Staphylococcus aureus* and *Listeria monocytogenes*, with MIC and MBC values ranging from 0.125 to 0.25 µg/mL. Remarkably, menthone and pulegone demonstrated MIC = MBC = 0.125 µg/mL against *S. aureus* and *L. monocytogenes*, respectively. In antidiabetic assays, all tested compounds outperformed acarbose. Menthone showed the best inhibition with IC₅₀ values of 149.32 ± 4.16 and 108.39 ± 4.08 μg/mL for α-amylase and α-glucosidase, respectively. MPEO also displayed potent dermatoprotective activity, with IC₅₀ values of 56.23 ± 2.24 μg/mL (tyrosinase) and 90.36 ± 2.26 μg/mL (elastase), compared to quercetin. In silico analysis confirmed strong binding affinities of pulegone and menthone to target enzymes involved in glucose regulation. These findings highlight MPEO as a promising natural source of bioactive compounds with antibacterial, antidiabetic, and dermatoprotective properties, supporting its potential use in pharmaceutical and therapeutic applications.

## Introduction

Despite significant medical progress, many chronic and infectious diseases continue to cause serious health and economic burdens worldwide. Among these, diabetes mellitus (DM) remains a major concern due to its growing prevalence (Saleem et al. 2022). Over the past decade, the number of diabetic patients has markedly increased across both industrialized and developing countries (Singh 2023), placing immense pressure on healthcare systems. The World Health Organization (WHO) has projected nearly 299 million cases of diabetes by 2025 (Taha et al. 2025). In 2021, the International Diabetes Federation estimated that 537 million adults (aged 20–79), representing 10.5% of the global adult population, were affected by diabetes (Bergman et al. 2024). This number is expected to rise to 643 million by 2030 (Kumar et al. 2024) and 783 million by 2045, accounting for 12.2% of adults worldwide (Sun et al. 2022). In addition, 541 million people had impaired glucose tolerance in 2021, a figure projected to reach 730 million by 2045 (Sun et al. 2022).

Simultaneously, infectious diseases caused by pathogenic bacteria continue to pose a major public health threat (Varela et al. 2021). The growing ineffectiveness of conventional antibiotics, mainly due to overuse and misuse, highlights the urgent need for alternative therapeutic strategies (Liu et al. 2023c). Furthermore, oxidative stress is recognized as a contributing factor to skin damage through the deterioration of cellular DNA, reinforcing the need for protective agents (Liu et al. 2023b).

In this context, natural products offer promising prospects in addressing these health challenges (Lucas et al. 2023). Historically, they have significantly contributed to drug discovery, particularly in anti-inflammatory, antimicrobial, and anticancer therapies (Elshafie et al. 2023). Today, natural products are incorporated into various pharmacological and cosmeceutical formulations for neuroprotective (Cores et al. 2023), dermatological (Silva-Flores et al. 2023), antidiabetic (Mata et al. 2023), antibacterial (Liu et al. 2023a), and anticancer (Ali Reza et al. 2023) purposes, primarily due to their good tolerability (Yuan et al. 2023). In particular, essential oils (EOs) from lavender, rose, mint, chamomile, and tea tree are widely used in the cosmetic industry (Sarkic and Stappen 2018).

However, despite numerous studies, the precise bioactive components responsible for the biological effects of many EOs remain unclear. The lack of a clear correlation between EO activity and their main chemical constituents, as well as insufficient combined analyses of these components, raises critical questions: Are the observed effects attributable to major compounds, minor constituents, or synergistic interactions?

The *Lamiaceae* family, especially the *Mentha* L. genus, comprises several species and cultivars widely distributed across South Africa, Australia, and temperate Eurasia (Akram et al. 2023; Kadoglidou and Chatzopoulou 2023). Among them, mint is commonly used as a food flavoring and possesses well-established medicinal properties (Akram et al. 2023). In Morocco, *Mentha pulegium* (pennyroyal), a widespread species, is traditionally consumed as an herbal infusion (Domingues and Santos 2019). *M. pulegium* essential oil (MPEO) has demonstrated antimicrobial, anti-inflammatory, and anticancer properties (Baali et al. 2019; Hajlaoui et al. 2009; Messaoudi et al. 2021), and is also employed in aromatherapy (Khadraoui et al. 2014; Roé et al. 2005). In previous studies, our team highlighted its antioxidant, antibacterial, and antileishmanial activities (Bouyahya et al. 2017).

MPEO contains several key volatile constituents such as piperitone, piperitenone, *α*-terpineol, pulegone, menthone, isomenthone, and limonene (Boukhebti et al. 2011; Brahmi et al. 2016; El-Ghorab 2006; Mahboubi and Haghi 2008; Teixeira et al. 2012). The composition varies depending on geographical origin and extraction methods. Notably, pulegone, a compound present in MPEO, has been identified as hepatotoxic and uterotoxic (Miraj and Kiani 2016; Thorup et al. 1983), and may present carcinogenic risks at high doses (National Toxicology Program 2011).

Toxicity evaluation often involves insecticidal assays. MPEO has shown insecticidal activity against *Callosobruchus maculatus* (Vojoudi et al. 2014), Curculionidae species (Ainane et al. 2019), *Tribolium castaneum* (Faraji et al. 2020), citrus pests (Attia et al. 2022), and *Aphis gossypii* (Akbari and Armideh 2023). In mice, acute dose-dependent toxicity has been reported, including respiratory distress and liver damage (Verdes et al. 2011). Nevertheless, recent studies by Caputo et al. (Caputo et al. 2021) suggest that a safe therapeutic use of MPEO is possible with proper phytochemical characterization (Caputo et al. 2021; Silva et al. 2017). According to OECD guidelines, an oral LD_50_ above 5000 mg/kg indicates low acute toxicity (Ez-Zriouli et al. 2022).

Therefore, precise dose control, targeted application strategies, and further studies could support the safe and effective therapeutic use of MPEO.

This study builds upon our previous study (Bouyahya et al. 2017), in which MPEO was extracted, chemically characterized by gas chromatography-mass spectrometry (GC–MS), and evaluated for its antioxidant, antibacterial, and antileishmanial activities. Using the same oil and experimental approach, we now aim to compare the biological activities of MPEO with those of its two major components—menthone and pulegone. This study also introduces a new evaluation of their antidiabetic potential, not previously explored, to better understand the contribution of these compounds to MPEO’s overall bioactivity.

In this context, we conducted a comparative study between the EO of *M. pulegium* and its two major constituents, menthone and pulegone, both monoterpenoids (C_10_H_18_O and C_10_H_16_O) commonly found in *Mentha* species (Domingues and Santos 2019). Although these compounds have shown anticancer, anti-inflammatory, and analgesic properties (Charlet et al. 2021; Giovana et al. 2016; Maurya et al. 2019), their dermatoprotective effects remain underexplored.

The aim of the present work is to evaluate and compare the antibacterial, antidiabetic, and dermatoprotective activities of MPEO and its two main components. To elucidate the mechanisms underlying their bioactivities, we employ both in vitro assays and in silico molecular docking simulations targeting key enzymes such as α-amylase, α-glucosidase, and tyrosinase.

## Materials and methods

### Chemicals and reagents

We purchased the compounds pulegone, menthone, quercetin, and acarbose from Sigma-Aldrich (France). The α-glucosidase from *Saccharomyces cerevisiae* and the α-amylase from *Bacillus licheniformis* were also obtained from reliable commercial sources. Additional reagents were carefully selected from reputable suppliers. Mueller–Hinton agar was sourced from Biokar in Beauvais, France. All other chemicals used are of analytical grade.

### Plant collection and essential oil extraction

*M. pulegium* L. was collected in 2016 from its natural habitat in the northwest Moroccan province of Ouezzane, located at 34° 47′ 50" N and 5° 34′ 56" W. The specimen, identified as RAB04 and carefully stored in the Department of Moroccan Botany of the Scientific Institute of Rabat (SIR), was verified for authenticity. The samples were left to dry naturally at room temperature in a shaded area. Subsequently, the EOs were extracted using a Clevenger-style apparatus through hydrodistillation. After measuring the amount of oils collected, anhydrous sodium sulfate was used to dehydrate them, and they were then stored at 4 °C until needed.

### Chemical composition analysis

As previously noted, MPEO's chemical composition was analyzed using the GC–MS approach (Bouyahya et al. 2017). Compounds were identified by comparing their mass spectra to the spectra in the Adams libraries (NIST/EPA/NIH, 2002; Adams, 2007) and the NIST02 library for the GC–MS system. Each element was confirmed on these references.

## In vitro biological activities

### Antidiabetic activity

#### α-Amylase inhibitory assay

We investigated the inhibitory potential of pulegone and menthone on α-amylase by exposing varying quantities of these substances to a reaction with the enzyme and a starch solution (Omari et al. 2023). Specifically, 250μL of the sample was mixed with an equal volume of α-amylase (240 U/mL) in a 0.02 M sodium phosphate buffer (SPB) (pH = 6.9). The mixtures were incubated at 37 °C for twenty minutes. Subsequently, 250 µL of a 1% starch solution in a 0.02 M SPB (pH = 6.9) was added to the reaction mixture, and the combination was allowed to sit at 37 °C for 15 min. One milliliter of dinitrosalicylic acid (DNS) was then introduced, and the blend was brought to a boil in a water bath for ten minutes. The reaction mixture was subsequently diluted with 2 mL of distilled water, and the absorbance was measured at 540 nm using an ultraviolet–visible spectrophotometer. Acarbose was utilized as a positive control to validate the findings as an α-amylase inhibitor.

#### α-Glucosidase inhibitory assay

Using the *p*-nitrophenyl α-_D_-glucopyranoside (pNPG) substrate, the inhibitory action of menthone and pulegone was investigated with minor adjustments based on the technique outlined in our previous study (Omari et al. 2023). Initially, 200 µL of the samples were mixed with 100 µL of 0.1 M SPB (pH = 6.7) containing 0.1 U/mL of α-glucosidase enzyme. The mixture was then incubated at 37 °C for ten minutes. One milligram of pNPG was dissolved in 0.1 ml of SPB (pH = 6.7). The activity of α-glucosidase was assessed by measuring the absorbance at 405 nm following the addition of 1 mL of 0.1 M Na_2_CO_3_ to the reaction mixture. The inhibitory action of menthone and pulegone was represented by the inhibition percentage, and IC_50_ values were calculated. Acarbose was used as a positive control.

### Dermatoprotective activity

#### Tyrosinase inhibitory assay

Using the method outlined by Marmouzi et al. (Marmouzi et al. 2019), the inhibitory ability of pulegone and menthone on tyrosinase was used to assess their dermatoprotective impact. In summary, 25 µL of sample was mixed with 100 µL of a tyrosinase solution (333 U/mL in 50 mM SPB, pH 6.5) and incubated for 10 min at 37 °C. After that, 300 µL of _L_-DOPA (5 mM) was added, and the mixture was kept at 37 °C for half an hour. The spectrophotometer was calibrated to detect absorbance at 510 nm. Tyrosinase inhibition rates were computed for EO dosages of 40, 60, 120, and 160 μg/mL, and IC_50_ values were determined. Quercetin was used as a positive control.

#### Elastase inhibitory assay

The objective of the study was to examine the inhibitory properties of pulegone and menthone on the activity of elastase, an essential enzyme involved in the breakdown of elastin present in skin and blood vessels. By adapting certain aspects, the methodology of this research is based on the protocol reported by Jeddi et al. (Jeddi et al. 2023).

To perform the experiment, varying amounts of pulegone and menthone were dissolved in methanol at concentrations of 0.5, 1, 2, and 3 mg/mL. Then, 200 µL of an elastase solution prepared in Tris–HCl buffer (0.2 M, pH 8.0) was mixed with 50 µL of each sample. Following a 15-min incubation at 25 °C, 200 µL of *N*-succinyl-Ala-Ala-p-nitroanilide solution was added, and the reaction mixtures were homogenized. Absorbances were recorded at 410 nm after an additional 20-min incubation at 25 °C. The data obtained were used to determine the extract concentration required to achieve 50% enzyme inhibition (IC_50_) and to calculate the percent inhibition of elastase. Quercetin was used as a positive control to validate the experimental design. This study provides important insights into the potential benefits of pulegone and menthone in inhibiting elastase activity, paving the way for promising applications in the medical and cosmetic fields. Furthermore, the methodology described in this study should provide a valuable resource for future research assessing the effect of other natural compounds on elastase activity, thereby expanding our understanding of their beneficial properties.

## Antibacterial activity

### Chemicals and reagents

We purchased the compounds menthol, carvacrol, tricosane, quercetin, and acarbose from Sigma-Aldrich (France). The α-glucosidase from *Saccharomyces cerevisiae* and the α-amylase from *Bacillus licheniformis* were also obtained from reliable commercial sources. Additional reagents were carefully selected from reputable suppliers. Mueller–Hinton agar was sourced from Biokar in Beauvais, France. All other chemicals used are of analytical grade.

### Bacterial strains

Four bacterial strains, comprising both Gram-negative and Gram-positive bacteria, were selected to assess the antibacterial activity of pulegone and menthone: *Staphylococcus aureus* ATCC 29213, *Escherichia coli* ATCC 25922, *Listeria monocytogenes* ATCC 13932, and *Pseudomonas aeruginosa* ATCC 27853.

### MIC and MBC determination

As mentioned before, the minimum inhibitory concentration (MIC) was established using the broth microdilution method. The minimum sample concentration required to destroy the bacteria is indicated by the minimum bactericidal concentration (MBC). The microdilution method, based on MIC determination, was similarly applied. After 24 h of incubation at 37 °C, 10 mL of sample from each tube showing no apparent growth were inoculated on Tryptone Soja agar (Biokar, Beauvais, France). The MBC was determined as the lowest concentration showing no growth on the culture medium.

## In silico biological activities

### Molecular modeling

The protein crystal structures of α-glucosidase (PDB ID: 3TOP) and α-amylase (PDB ID: 1HNY) were obtained from the Protein Data Bank. Additionally, homology models for human tyrosinase and elastase were constructed using the crystal structure of mushroom tyrosinase (PDB ID: 2Y9X) and the protein structure of porcine pancreatic elastase (PDB ID: 4YM9), respectively. All 3D structures of the ligands were downloaded from the PubChem database. In silico molecular docking simulations were performed using the AutoDock Vina program. The processes of hydrogenation, particle charge distribution, and fusion of nonpolar hydrogen atoms were carried out using Autodocktools-1.5.7 software. The dimensions of the grid boxes were defined for each enzyme as follows: a 40 Å × 16 Å × 60 Å box centered at X = 3.889, Y = 49.335 and Z = 22.552 for α-amylase, a 52 Å × 40 Å × 50 Å box centered at X = −32.492, Y = 28.299 and Z = 29.8 for α-glucosidase, a 28 Å × 40 Å × 30 Å box centered at X = 11.03, Y = 8.605 and Z = 1.208 for the elastase simulations, and a 38 Å × 52 Å × 32 Å centered at X = −8.947, Y = −30.22 and Z = −43.477 for tyrosinase. Graphical visualization of all docking complexes was conducted using Discovery Studio 2024 Client software.

### Statistical analysis

All antioxidants, antidiabetic, and dermatoprotective assays were performed in triplicate to ensure the repeatability and reliability of the results. Data are presented as mean ± standard deviation (SD). Statistical analysis was conducted using GraphPad Prism (version 10.1). A two-way ANOVA followed by Tukey’s multiple comparison test was used to determine statistically significant differences between groups.

## Results

### Chemical composition of MPEO

GC–MS analysis was employed to determine the phytochemical content of MPEO. Our previous results revealed a diverse composition, primarily consisting of two major substances: pulegone (41.0%) and menthone (21.2%) (Bouyahya et al. 2017). These compounds were subsequently purchased and tested separately to ascertain the source of MPEO’s efficacy, as previously indicated by our research team (Bouyahya et al. 2017). Other compounds identified in MPEO include α-terpineol (8.0%), humulene (5.4%), eucalyptol (5.2%), verbenone (4.5%), 1,8-cineole (4.0%), and _D_-limonene (2.4%) (Table 1). This phytochemical diversity underscores the intrinsic complexity of this oil’s composition, demonstrating the richness and profound variability of its chemical constituents, thereby reinforcing the uniqueness of the EO extracted from *M. pulegium* in the Ouezzane region of Morocco.

**Table 1:** Chemical composition of *M. pulegium* essential oils (Bouyahya et al. 2017)

Compounds	*Mentha pulegium*	Identification
*α*-pinene	0.536	MS, IR
Camphene	0.447	MS, IR
Sabinene	0.746	MS, IR
*β*-pinene	0.172	MS, IR
1-Octen-3-ol	0.473	MS, IR
3-Octanone	0.433	MS, IR
*p*-cymene	0.218	MS, IR
_D_-Limonene	2.422	MS, IR
Eucaliptol	5.199	MS, IR
1,8-Cineole	3.82	MS, IR
*α*-Terpineol	7.98	MS, IR
Verbenone	4.535	MS, IR
Menthone	21.164	MS, IR
Neo-menthol	2.272	MS, IR
Menthol	1.494	MS, IR
Isopulegone	0.187	MS, IR
Pulegone	40.98	MS, IR
Humulene	5.402	MS, IR
caryophyllene oxide	0.263	MS, IR

## In vitro biological activities of MPEO, menthone, and pulegone

### Antidiabetic effects

DM represents a major endocrine metabolic disorder characterized by an abnormal elevation of blood glucose levels. This elevation is primarily attributed to two key processes: the breakdown of starch by pancreatic α-amylase and the subsequent absorption of glucose by α-glucosidase in the small intestine (Souza et al. 2012). Accordingly, inhibition of these two enzymes could be pivotal in regulating postprandial glycemia, potentially curbing the progression of diabetes. This targeted therapeutic approach offers a promising prospect in DM management, aiming to maintain more stable blood glucose levels and alleviate complications associated with this condition.

The α-glucosidase and α-amylase inhibitory capacities of MPEO, menthone, and pulegone were evaluated. The findings are presented in Table 2 and compared to those of acarbose, used as a positive control. The results showed that menthone presents promising inhibitory potency towards both enzymes, with IC_50_ values of 108.39 ± 4.08 and 149.32 ± 4.16 µg/mL for α-glucosidase and α-amylase, respectively. In contrast, MPEO and pulegone revealed less inhibitory activity compared to menthone, displaying IC_50_ values of 251.18 ± 6.26 and 182.41 ± 5.58 µg/mL, respectively, for α-amylase, as well as 113.42 ± 2.43 and 119.15 ± 5.26 µg/mL for α-glucosidase. Moreover, this activity remains more important than that of acarbose, with an IC_50_ of 199.53 ± 3.26 µg/mL against α-glucosidase and 396.42 ± 4.83 µg/mL against α-amylase. Thus, MPEO shows notable inhibitory activity against both enzymes.

**Table 2:** Inhibitory activity of MPEO, menthone, and pulegone against antidiabetic enzymes (α-amylase and α-glucosidase) (IC_50_ in μg/mL)

	α-amylase	α-glucosidase
Menthone	149.32 ± 4.16^c^	108.39 ± 4.08^b^
Pulegone	182.41 ± 5.58^c^	119.15 ± 5.26^b^
MPEO	251.18 ± 6.26^a^	113.42 ± 2.43^b^
Acarbose	396.42 ± 4.83^a^	199.53 ± 3.26^a^

Different superscript letters in the same columnindicate statistically significant differences (*p* < 0.05), whereas identical letters indicate no significant difference

### Dermatoprotective effects

Skin aging is a complex biological process influenced by various endogenous factors such as genetic, hormonal, and cellular metabolic aspects, as well as exogenous factors like exposure to radiation, chemicals, and environmental pollution. This process leads to the accumulation of ROS and the activation of skin enzymes, including elastase and tyrosinase, which are associated with various dermatological disorders. Indeed, this work aims to evaluate the ability of MPEO and its two main components, menthone and pulegone, to protect the skin using tyrosinase and elastase inhibition assays. The results are expressed in terms of IC_50_ and compared to those of quercetin, used as a positive control.

As noted, all samples demonstrated inhibition of _L_-DOPA oxidation by tyrosinase, as shown in Table 3. However, MPEO displayed the best activity, with an IC_50_ value of 56.23 ± 2.24 μg/mL, compared to menthone (217.14 ± 6.12 μg/mL) and pulegone (239.75 ± 4.28 μg/mL). These results confirm the inhibitory effect of MPEO, attributable to its high contents of these compounds and their synergistic action. Furthermore, MPEO and its two main components displayed greater inhibitory activity than quercetin, which had an IC_50_ value of 246.90 ± 2.54 μg/mL.

**Table 3:** Inhibitory activity of MPEO, menthone, and pulegone against dermatoprotective enzymes (tyrosinase and elastase) (IC_50_ in μg/mL)

	Tyrosinase	Elastase
Menthone	217.14 ± 6.12^a^	132.72 ± 4.27^a^
Pulegone	239.75 ± 4.28^a^	117.84 ± 3.88^a^
MPEO	56.23 ± 2.24^c^	90.36 ± 2.26^a^
Quercetin	246.90 ± 2.54^a^	9.08 ± 0.21^c^

Different superscript letters in the same columnindicate statistically significant differences (*p* < 0.05), whereas identical letters indicate no significant difference

For anti-elastase activity, similar results were observed with MPEO and its major components. In fact, MPEO demonstrated the strongest inhibitory activity, with an IC_50_ value of 90.36 ± 2.26 μg/mL, compared to menthone (132.72 ± 4.27 μg/mL) and pulegone (117.84 ± 3.88 μg/mL). Additionally, quercetin, a potent elastase inhibitor, exhibited the best enzyme inhibition, with an IC_50_ value of 9.08 ± 0.21 μg/mL compared to MPEO and its lead compounds. These findings indicate that the synergistic effect between menthone, pulegone, and other components present in the EO is responsible for the significant inhibitory effects of MPEO on both elastase and tyrosinase.

### Antibacterial activity

In this research, we examined the antibacterial activities of MPEO and its main compounds using the broth microdilution method. We evaluated their effectiveness against Gram-negative bacteria (*E. coli* and *P. aeruginosa*) and against Gram-positive bacteria (*L. monocytogenes* and *S. aureus*).

Our current study demonstrated significant antibacterial activity of MPEO and its two compounds against all tested pathogens. The MIC and MBC values, as presented in Table 4, indicate variation in activity depending on species. We observed that strains of *S. aureus* and *L. monocytogenes* were particularly sensitive to the EOs of *M. pulegium* and its major compounds, with MIC values ranging between 0.125 and 0.5 µg/mL. In contrast, *P. aeruginosa* and *E. coli* showed less sensitivity to the EOs and to both compounds, with MIC values ranging from 0.5 to 2 µg/mL. Interestingly, MPEO showed a strong inhibitory effect against the Gram-positive bacteria tested.

**Table 4:** The MBC and MIC values (in µg/mL) of MPEO, pulegone, and menthone

Molecules	MIC/MBC	*Escherichia coli*	*Pseudomonas aeruginosa*	*Staphylococcus aureus*	*Listeria monocytogenes*
Menthone	MIC	1	0.5	0.125	0.125
	MBC	> 2	1	0.125	0.25
Pulegone	MIC	0.25	0.5	0.125	0.125
	MBC	0.5	0.5	0.25	0.125
MPEO	MIC	0.5	2	0.25	0.5
	MBC	1	2	0.25	0.5
Chloramphenicol	MIC	8	64	8	16
	MBC	32	64	32	32

## In silico biological activities of mpeo, menthone, and pulegone

### Molecular modeling

This research aimed to explore the molecular interactions between the main phytochemical compounds (menthone and pulegone) in MPEO and key enzymes, including α-glucosidase, α-amylase, elastase, and tyrosinase. The docking scores for menthone and pulegone with these enzymes are presented in Table 5.

**Table 5:** Binding affinities of menthone and pulegone with the protein structures of α-amylase, α-glucosidase, elastase, and tyrosinase (in kcal/mol)

Bioactive compounds	Antidiabetic effects		Dermatoprotective effects	
	α-glucosidase	α-amylase	Tyrosinase	Elastase
Menthone	− 6.0	− 5.7	− 5.3	− 4.5
Pulegone	− 6.9	− 5.8	− 5.9	− 4.8
Standards	Acarbose		Quercetin	
	− 7.2	− 6.0	− 7.7	− 6.7

**Fig. 1:**
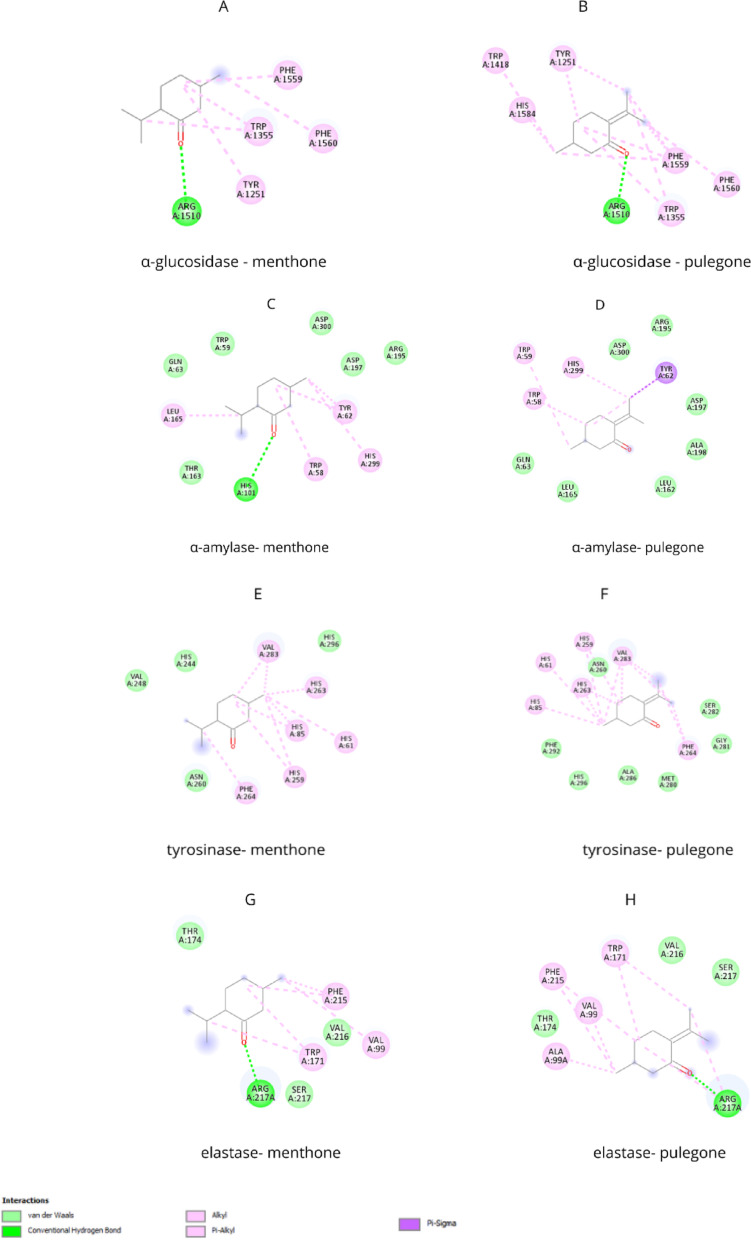
Protein–ligand interaction: **A** α-glucosidase and menthone, **B** α-glucosidase and pulegone, **C** α-amylase and menthone, **D** α-amylase and pulegone, **E** tyrosinase and menthone, **F** tyrosinase and pulegone, **G** elastase and menthone, **H** elastase and pulegone

For α-glucosidase, menthone exhibited a binding affinity (BA) of − 6.0 kcal/mol, whereas pulegone displayed a higher BA of − 6.9 kcal/mol, approaching the reference value of acarbose at − 7.2 kcal/mol. Both compounds were observed binding to the active site of α-glucosidase, forming a hydrogen bond with Arg1510 and interacting with several hydrophobic residues, such as Phe1559, Phe1560, Trp1355, and Tyr1251 (Fig. 1A, B). Similarly, menthone and pulegone exhibited significant interactions with α-amylase. The docking energies for both compounds were nearly identical, with pulegone at − 5.8 kcal/mol and menthone at − 5.7 kcal/mol, compared to acarbose at − 6.0 kcal/mol. Key interactions for α-amylase included a hydrogen bond between menthone and HIS101, as well as several hydrophobic contacts involving Tyr62, His299, Trp58, and Leu165 (Fig. 1C). Likewise, pulegone established Pi-Sigma interactions with Tyr62 and hydrophobic contacts with Trp59, Trp58, and His299, along with multiple van der Waals interactions (Fig. 1D). These results suggest that pulegone, in particular, could be a promising candidate for antidiabetic treatments.

Regarding dermal pigmentation and remodeling enzymes, both menthone and pulegone occupied the tyrosinase catalytic site, albeit with lower BAs than quercetin. Menthone showed a BA of − 5.3 kcal/mol, whereas pulegone had a higher BA of − 5.9 kcal/mol, compared to quercetin’s − 7.7 kcal/mol. The interactions between pulegone, menthone, and the catalytic pocket involved hydrophobic bonds with specific residues, such as His61, His85, His263, His259, Val283, and Phe264, reinforced by additional van der Waals interactions (Fig. 1E, F).

For elastase, pulegone and menthone exhibited affinities of − 4.8 and − 4.5 kcal/mol, respectively. Their primary interactions included a hydrogen bond with Arg217 and hydrophobic contacts with Ala99, Val99, Phe215, and Trp171 (Fig. 1G, H). These findings indicate that both menthone and pulegone may have potential dermatological applications for pigmentation control and skin remodeling, with pulegone showing stronger interactions.

## Discussion

The chemical composition of MPEO has been analyzed in previous studies conducted in various regions of Morocco and other countries (Agnihotri et al. 2005; Bekka-Hadji et al. 2022; Bektašević et al. 2021; Chraibi et al. 2016; Derwich et al. 2010; Lorenzo et al. 2002; Ouakouak et al. 2015; Oualdi et al. 2023; Pino et al. 1996; Sbayou et al. 2014; Stoyanova et al. 2005; Yasa et al. 2012; Zanjani et al. 2015). The results of these studies largely align with our conclusions, revealing minimal variability. The chemotaxonomy of *M. pulegium* is influenced not only by the species but also by various other elements, including phenological factors, geographic variations, extraction methods, edaphic characteristics, and the climatic conditions specific to the harvest location (Ahmed et al. 2018; Zantar et al. 2015b, 2015a). The interaction of these factors can result in substantial changes in a plant’s chemovariability. It is noteworthy that this complex chemical composition has generated considerable interest, leading to numerous research efforts aimed at highlighting its biological properties. In this context, menthone and pulegone were selected for an in vitro and in silico study of their biological activity in conjunction with MPEO (Table 1).

Notably, research on the antidiabetic activity of *M. pulegium* remains limited. Abbou et al. (2022) reported that the ethyl acetate fraction of its aerial parts exhibited strong inhibitory activity against α-glucosidase and α-amylase, with IC_50_ values of 61.85 ± 1.69 µg/mL and 16.37 ± 0.11 µg/mL, respectively, more effective than acarbose (IC_50_ = 31.03 ± 1.98 and 150.26 ± 0.88 µg/mL). Similarly, Gülçin et al. (2020) found that methanolic and aqueous extracts inhibited both enzymes, with IC_50_ values of 20.38 and 21.65 µM for α-glucosidase, and 23.11 and 36.47 µM for α-amylase. In vivo, Farid et al. (Farid et al. 2019) demonstrated that oral administration of *M. pulegium* (20 mg/kg) significantly improved glucose tolerance and exhibited antihyperglycemic and antihypercholesterolemic effects in streptozotocin (STZ)-induced diabetic rats. Variations in outcomes likely reflect differences in experimental protocols, biological variability, extract composition, and methodological biases. In addition to digestive enzyme inhibition, several other biological activities play a promising role in the management and treatment of diabetes, including antioxidant and anti-inflammatory activities. These complementary mechanisms may contribute to better glycemic control and prevention of complications associated with the disease. Chronic inflammation is closely related to insulin resistance and the progression of T2DM. Indeed, anti-inflammatory agents reduce the production of pro-inflammatory cytokines, thereby improving insulin sensitivity and metabolic function.

Given that protein denaturation is one of the main causes of inflammation (Silvestrini and Silvestrini 2023), Luís and Domingues (Luís and Domingues 2021) evaluated the ability of MPEO to inhibit this process. Their results showed that this oil has an inhibitory capacity (IC_50_ = 88.31 ± 1.37 (%, *v*/*v*)) very similar to that of acetylsalicylic acid (IC_50_ = 89.47 ± 2.64 (%, *v*/*v*)), a well-known anti-inflammatory agent. These values indicate that MPEO has significant potential as an anti-inflammatory agent, comparable to that of acetylsalicylic acid.

Furthermore, a very recent study examined the anti-inflammatory potential of MPEO, thyme honey (TH), and their mixtures, both in vivo and in vitro (Assaggaf et al. 2024). In vivo, the anti-inflammatory effect was evaluated on carrageenan-induced inflammatory edema in the left paw of rats. In vitro, the inhibition of 5-Lipoxygenase (5-LOX) was analyzed. The results showed that, whether in vivo or in vitro, MPEO alone or in combination with TH reduced inflammation. The maximum efficacy was observed with the combination of both, suggesting a potential synergy between MPEO and TH.

Given the pivotal role of oxidative stress in the development of T2DM, antioxidant activity is particularly relevant for its prevention and management (Papachristoforou et al. 2020). MPEO from the Ouazzane region has shown notable antioxidant potential, with IC_50_ values of 58.27 μg/mL (FRAP) and 321.41 μg/mL (DPPH) (Bouyahya et al. 2017), and a total antioxidant capacity of 583.07 mg EAA/g EO (Aimad et al. 2021). Comparable studies in Algeria (Abdelli et al. 2016; Baali et al. 2019; Brahmi et al. 2016), Iran (Kamkar et al. 2010; Nickavar and Jabbareh 2018), Egypt (El-Ghorab 2006), and Turkey (Sarikurkcu et al. 2012) highlight geographic variability. The strong antioxidant capacity of MPEO from Ouazzane may support beta cell protection, enhance insulin sensitivity, and help manage oxidative complications in diabetes.

The effectiveness of this EO can be attributed to its high concentration of menthone (21.2%) and pulegone (41.0%), as well as their synergistic interactions with other compounds present in lower quantities (menthol, verbenone, pinene, limonene, camphene, *p*-cymene, etc.). In fact, the potential antidiabetic activity of menthone was investigated in vitro on insulin-resistant 3T3-L1 adipocytes. The results show that the menthone at a dose of 63.22 µM/mL effectively reduced insulin resistance by enhancing glutathione peroxidase (GPx) and catalase enzyme activity and reactive oxygen species (ROS) reduction in mature 3T3L-1 adipocyte cells (Revathi et al. 2022). On the other hand, Muruganathan et al. (2017) investigated the effect of menthol, present as a minor compound in our study, on glucose metabolic enzymes and apoptosis of pancreatic islet cells in STZ–nicotinamide-induced diabetic rats. The treatment, administered at various doses over a period of 45 days, revealed a significant reduction in blood glucose and glycosylated hemoglobin (HbA1c) levels, as well as an increase in plasma insulin and hepatic glycogen levels. Menthol also restored the activities of altered glucose metabolic enzymes and serum biomarkers of liver damage, thereby mitigating pathological anomalies in the pancreatic islets of diabetic rats by inhibiting β-cell apoptosis. Tijjani et al. (2022) investigated the antihyperglycemic effects of verbenone both in vitro and in vivo. In vitro, verbenone demonstrated significant inhibitory activity against α-amylase and α-glucosidase. In vivo, it significantly decreased blood glucose levels in treated diabetic mice. The same results were obtained in diabetic rats treated with β-pinene (Santos et al. 2022). Another isomer of pinene, α-pinene, has shown promising hypoglycemic activity, whether administered alone or in combination with antidiabetic drugs. Özbek and Sever Yilmaz (2017) and Rafieirad et al. (2022) observed this effect in diabetic mice and rats, respectively. Our research team previously conducted a study evaluating this potential on two digestive enzymes, namely α-amylase and α-glucosidase, and recorded IC_50_ values of 82.12 ± 4.52 and 95.62 ± 4.11 μg/mL, respectively (Omari et al. 2023). Additionally, Feriotto et al. (Feriotto et al. 2024) investigated the effects of α-pinene on the expression and translocation of the glucose transporter-4 (GLUT4) in murine muscle cells, thereby mimicking the effect of insulin. Another organic compound, limonene, has demonstrated significant antidiabetic effects, either alone or in combination with other substances. More et al. (More et al. 2014) combined limonene with linalool for oral treatment of STZ-induced diabetic rats, observing inhibition of protein glycation and a reduction in blood glucose levels. Shakeel and Tabassum (Shakeel and Tabassum 2022) also showed an improvement in glycemic parameters in T2D rats following daily administration of _D_-limonene. Moreover, EL Hachlafi et al. (Hachlafi et al. 2023) reported that camphene exerts its antidiabetic action through various mechanisms, such as increasing the translocation of GLUT2, enhancing the transcription of cytochrome P450 2 (CUP2) and uncoupling protein 1 (UCPCUP1) genes, decreasing the expression of peroxisome proliferator-activated receptor gamma (PPAR-γ) and CCAAT/enhancer-binding protein alpha (C/EBPα), and stimulating the adiponectin and AMP-activated protein kinase (AMPK) signaling pathways. Furthermore, *p*-cymene has demonstrated antidiabetic effects in several studies (Balahbib et al. 2021). In fact, Lotfi et al. (Lotfi et al. 2015) evaluated the effect of this terpene on blood glucose levels in mice fed a high-fat diet, finding a significant reduction in glycemia. Abbasi et al. (2018) investigated the anti-glycation effect of *p*-cymene in vitro, observing a significant reduction in the formation of advanced glycation end-products.

The antidiabetic effect of MPEO appears to be attributed not only to its major compounds, menthone and pulegone, but also to the synergy with various minor compounds such as menthol, verbenone, pinene, limonene, camphene, and *p*-cymene. These compounds, although present in smaller quantities, can significantly contribute to the overall therapeutic effect through various biochemical and physiological mechanisms.

These findings suggest that the use of MPEOs and their components could provide considerable benefits in modulating the rate of carbohydrate digestion and absorption. This therapeutic approach could be instrumental in controlling hyperglycemia associated with diabetes. These findings pave the way for new research perspectives on the potential use of natural compounds from *M. pulegium* in the development of effective treatments for diabetes.

Regarding the dermatoprotective activity of MPEO, it has attracted interest due to its bioactive compounds that may interact with key skin enzymes. Tyrosinase, involved in melanin synthesis, catalyzes the initial steps of melanogenesis and plays a central role in skin, hair, and eye pigmentation (Chiocchio et al. 2018; Kanlayavattanakul and Lourith 2018; Zheng et al. 2010). Its misregulation can lead to pigmentation disorders, such as urticaria pigmentosa and age-related skin changes (Gonçalves et al. 2017), making tyrosinase inhibition a promising strategy to reduce melanin overproduction.

Similarly, elastase, a chymotrypsin-like protease, degrades elastin, essential for skin elasticity, and its overactivity contributes to skin aging, including wrinkles and sagging (Chiocchio et al. 2018; Zar Wynn Myint et al. 2021). Inhibiting elastase thus supports the preservation of skin structure and elasticity.

Consequently, targeting tyrosinase and elastase is a key dermatological strategy to combat hyperpigmentation and aging. Many skincare products incorporate enzyme inhibitors to maintain healthy, youthful skin by regulating melanin production and protecting the extracellular matrix (Jiratchayamaethasakul et al. 2020).

To the best of our knowledge, there is only one recent study that has evaluated the anti-tyrosinase activity of MPEO (Assaggaf et al. 2024). However, no prior research has investigated its anti-elastase effect, making our results innovative in this field. This highlights a significant gap in the literature, underscoring the need for further studies to explore the potential multifaceted skin-related benefits of MPEO, particularly its role in inhibiting elastase activity, which is important for maintaining skin elasticity and preventing aging. Indeed, Assaggaf et al. (Assaggaf et al. 2024) investigated the dermatoprotective effect of MPEO combined with TH, showing a promising synergistic inhibition of tyrosinase (IC_50_ = 53.96 ± 0.05 mg/mL), compared to quercetin (IC_50_ = 25.41 ± 0.06 mg/mL). As a bifunctional enzyme involved in phenol oxidation and melanin synthesis, tyrosinase is a common target in skincare. These results support the potential of MPEO-TH combinations in regulating melanin production**.** Similarly, Fatiha et al. (Fatiha et al. 2015) reported notable tyrosinase inhibition by an ethanolic extract of *M. pulegium* (IC_50_ = 286 ± 45 µg/mL). Regarding elastase, MPEO exhibited strong inhibitory activity (IC_50_ = 90.36 ± 2.26 µg/mL), outperforming menthone (132.72 ± 4.27 µg/mL) and pulegone (117.84 ± 3.88 µg/mL), though quercetin remained the most potent (IC_50_ = 9.08 ± 0.21 µg/mL). This suggests a synergistic effect among MPEO constituents**.** Additionally, our previous study showed that limonene and α-pinene, two minor components of *M. pulegium*, demonstrated notable anti-tyrosinase (IC_50_ = 74.24 ± 2.06 and 97.45 ± 5.22 μg/mL) and anti-elastase activities (IC_50_ = 91.25 ± 3.06 and 64.18 ± 2.80 μg/mL), though quercetin remained more effective against elastase.

Our results suggest that MPEO, menthone, and pulegone may inhibit enzymatic activity, potentially reducing melanin secretion. However, quercetin remains the most potent elastase inhibitor among the tested compounds, warranting further preclinical studies. Although studies assessing the dermatoprotective effects of this plant via enzyme inhibition are scarce, some have reported its potential in enhancing wound healing and protecting skin from damage (Baali et al. 2022; Khezri et al. 2020). There is a strong link between wound healing capacity and dermatoprotection, as maintaining healthy skin supports faster lesion repair. Certain compounds not only promote healing (e.g., by boosting collagen synthesis or reducing inflammation) but also reinforce the skin barrier against environmental stressors like UV radiation and pollutants (Khezri et al. 2020; Messire et al. 2023; Michalak et al. 2021; Mohd Zaid et al. 2022). Pulegone, in fact, has shown wound healing effects in rats, with increased hydroxyproline levels and improved biomechanical properties such as yield strength and stiffness (Cheraghali et al. 2017). It also alleviated atopic dermatitis symptoms in vivo by inhibiting nuclear factor kappa B (NF-κB) and mitogen-activated protein kinase (MAPK) signaling and cytokine production (Choi et al. 2018). As for α-pinene, Fraternale et al. (Fraternale et al. 2019) demonstrated its inhibitory effect on elastase activity, alone or combined with limonene, though less potent than the reference molecule.

Additionally, Karthikeyan and colleagues conducted two consecutive studies to evaluate the protective effects of α-pinene against DNA damage, inflammation, and UVA-induced skin damage in human (Karthikeyan et al. 2018) and mouse skin (Karthikeyan et al. 2019). In human keratinocytes, α-pinene reduced cytotoxicity, modulated nucleotide excision repair (NER) proteins, and inhibited inflammatory mediators. In mice, it inhibited lipid peroxidation, enhanced angiogenesis, and suppressed matrix metalloproteinase (MMP) and apoptotic gene expression, thus preventing dermal tissue damage. Regarding limonene, Kulig et al. (Kulig et al. 2022) reported significant anti-tyrosinase activity, while Uddin (Uddin 2014) showed that oral _D_-limonene treatment prevented sunburn and reduced cell proliferation in UV-exposed mouse skin.

Overall, the dermatoprotective potential of MPEO is strongly linked to its major compounds (menthone and pulegone), as well as to minor ones like limonene and α-pinene, and the synergy between them. This combination results in potent inhibition of enzymes like tyrosinase and elastase, which are associated with skin aging and pigmentation. These findings highlight MPEO’s potential as a valuable ingredient in cosmetic formulations aimed at treating skin conditions and promoting skin health.

Given that many cosmetic products now incorporate enzyme-inhibiting agents for anti-wrinkle, skin-lightening, and anti-aging effects, MPEO and its compounds could be considered promising candidates for addressing hyperpigmentation, uneven skin tone, wrinkles, and signs of premature or intrinsic aging.

Regarding the antibacterial activity of MPEO, our results confirm its effectiveness, particularly against Gram-positive bacteria. Our findings are in full agreement with previous studies, which reported that Gram-positive bacteria are more sensitive to MPEOs than Gram-negative bacteria. Indeed, Baali et al. (Baali et al. 2019) studied the effect of MPEO against several bacterial strains (including five Gram-positive and five Gram-negative) and reported that MPEO exhibits remarkable antibacterial properties, with the lowest MIC values observed against *Bacillus subtilis* (0.15 ± 0.01 mg/mL) and *S. aureus* (0.30 ± 0.01 mg/mL). Another study on pennyroyal EO recorded moderate activity against *S. aureus* (MIC = 1.406 µg/mL) and *B. subtilis* (MIC = 2.812 µg/mL) (Aimad et al. 2021). Ait-Ouazzou et al. (Ait-Ouazzou et al. 2012) investigated the antibacterial properties of MPEO against Gram-positive bacteria (*Enterococcus faecium*, *S. aureus*, and *L. monocytogenes*) and Gram-negative bacteria (*Salmonella enteritidis*, *E. coli*, and *P. aeruginosa*). Their results revealed bacteriostatic activity on all strains tested, with notable effectiveness against *E. faecium* (MIC < 0.5 µg/mL), *L. monocytogenes* (MIC = 1 µg/mL), and *S. aureus* (MIC = 1 µg/mL). The sensitivity of Gram-positive bacteria to EO is explained by the structure of their cell wall, known to promote their receptivity to this type of compound. In contrast, the increased resistance of Gram-negative bacteria can be attributed to the complexity of their cell wall, which limits the penetration of hydrophobic compounds through their lipopolysaccharide envelope (Ait-Ouazzou et al. 2012; Jebali et al. 2022; Messaoudi et al. 2021). Furthermore, several investigations have evaluated the antibacterial potential of MPEO from various regions around the world on several pathogenic bacterial strains (Amalich et al. 2016; Dehghani et al. 2018; Jazani et al. 2009; Mahboubi and Haghi 2008; Marzouk et al. 2008; Morteza-Semnani et al. 2011; Rahmani et al. 2018; Silva et al. 2015; Teixeira et al. 2012). These studies have followed a similar experimental approach to ours, using the disk/well diffusion technique on agar to estimate the bacteriostatic activity of EOs and their major compounds. This method involves measuring the zone of inhibition of bacterial growth around the disks or wells impregnated with the EO. Additionally, the liquid broth dilution method has been employed to determine the MIC and MBC. However, some studies have evaluated the antibacterial effect of MPEO using only the disk diffusion method (Boukhebti et al. 2011) or the liquid broth dilution method (Gourich et al. 2022; Shahmohamadi et al. 2011), while our comprehensive approach combined both techniques to provide a more robust assessment. The findings of all these studies confirm the antibacterial effect of MPEO against various pathogenic bacteria. Researchers have reported significant inhibition zones and microdilution results confirming the antibacterial efficacy of MPEO, offering valuable insights for therapeutic applications. Rahchamani et al. (Rahchamani et al. 2023) evaluated MPEO’s activity using MIC, MBC, and time-kill assays against *Streptococcus agalactiae*, *E. coli*, and *S. aureus* (three strains linked to bovine mastitis) and observed a marked reduction in bacterial counts within 24 h. Chraibi et al. (Chraibi et al. 2021) showed a synergistic effect when MPEO was combined with *M. piperita* EO, especially against *S. aureus* and *E. coli*. Similarly, Cherrat et al. (Cherrat et al. 2014) demonstrated enhanced bactericidal effects when MPEO was combined with mild heat, pulsed electric fields (PEF), or high hydrostatic pressure (HHP) at pH 4.0 and 7.0, achieving reductions of up to 5 log_10_ cycles. The antibacterial effects of MPEO are partly attributed to its anti-biofilm (Barchan et al. 2016; Tutar et al. 2016) and anti-quorum sensing (QS) properties (Luís and Domingues 2021). Biofilms, complex bacterial communities embedded in a protective matrix (Zhao et al. 2023a), are highly resistant to antimicrobials and immune responses (Costerton et al. 1995). By disrupting biofilm formation, MPEO weakens bacterial persistence. Additionally, its components interfere with QS signaling (Rattray et al. 2022), inhibiting coordinated behaviors such as virulence, antibiotic resistance, and biofilm development, key processes in bacterial pathogenesis.

In contrast, the phytochemical analysis of MPEO revealed pulegone (41.0%) and menthone (21.2%) as major components, which may act synergistically with minor compounds to enhance antibacterial efficacy. Pulegone, in particular, has demonstrated notable antibacterial activity against various multidrug-resistant strains (Amalich et al. 2016; Baygar 2019; Farhanghi et al. 2022; Gong et al. 2021; Nozohour and Jalilzadeh-amin 2021; Shahdadi et al. 2023; Sonboli et al. 2006). Sonboli et al. (2006) reported significant activity against *S. aureus* and *S. epidermidis* (1.8 ± 0.3 mg/mL), while Amalich et al. (Amalich et al. 2016) showed its inhibitory effects on *P. aeruginosa*, *S. aureus*, and *K. pneumoniae* (2.80–11.20 µl/mL). Baygar (2019) found strong activity against *S. maltophilia* (28 mm inhibition zone, MIC = 2.5 mg/mL, MBC = 5 mg/mL), a multidrug-resistant bacterium. Pulegone also inhibited biofilm formation and showed efficacy against *Streptococcus equi* subsp. *equi* (20 mm inhibition zone) (Nozohour and Jalilzadeh-amin 2021). Against *E. coli* K1, it reduced biofilm formation by 52.36%, with MIC and MBC values of 23.68 and 47.35 mg/mL, respectively, and downregulated *pgaABCD* gene expression involved in the synthesis of poly-β-1,6-*N*-acetyl-d-glucosamine (PNAG) (Gong et al. 2021).

Interestingly, when combined with eucalyptol, pulegone exhibited a different mechanism of action against *S. aureus* by inducing the release of bacterial cellular components, with MIC values of 23.43 μl/mL for eucalyptol and 5.85 μl/mL for pulegone (Farhanghi et al. 2022). Recently, Shahdadi et al. (Shahdadi et al. 2023) demonstrated that adding pulegone to edible coatings made of chitosan and alginate significantly reduced the growth of *S. aureus, L. monocytogenes*, and *E. coli* during cheese storage. Regarding menthone, Zhao et al. (Zhao et al. 2023b) investigated its antibacterial effect and mechanism against methicillin resistant *Staphylococcus aureus* (MRSA). According to this study, menthone exhibited a potent inhibitory and killing effect on MRSA, with reported MBC and MIC values of 7.1 and 3.5 μg/mL, respectively. The antibacterial mechanism involved the disruption of bacterial membrane integrity, membrane potential depolarization, and disturbance of lipid homeostasis in MRSA cells. Thus, MPEO and its major compounds show promise as alternatives to synthetic preservatives in the food industry and synthetic antibiotics, due to their considerable antimicrobial potential.

Overall, the antibacterial efficacy of MPEO and its main compounds is attributed to a combination of mechanisms, including inhibiting biofilm formation, interfering with QS, and directly disrupting bacterial cellular structures and functions. These properties make MPEO a promising natural therapeutic agent for combating difficult-to-treat bacterial infections, particularly those caused by antibiotic-resistant strains.

On the other hand, a molecular docking study was conducted to better understand the antidiabetic and dermatoprotective properties of menthone and pulegone, the main compounds in MPEO. This in silico approach estimates ligand affinity for enzymatic targets by predicting optimal conformations within protein binding sites (Raval and Ganatra 2022; Sahu et al. 2024). Commonly used in drug discovery, it involves evaluating flexible ligands with rigid proteins through advanced algorithms (Surana et al. 2021).

Our findings suggest pulegone is a promising antidiabetic agent due to its favorable interactions with α-glucosidase and α-amylase, while menthone, with slightly lower affinities, may also be useful. Pulegone also interacts with the catalytic site of gamma-aminobutyric acid transaminase (GABA-T), forming non-covalent interactions (Abdelgaleil et al. 2019). Since GABA modulates insulin secretion and glucose regulation (Dong et al. 2006; Purwana et al. 2014), GABA-T inhibition may offer glycemic benefits.

Regarding dermatological effects, both pulegone and menthone bind to tyrosinase and elastase, though with lower affinity than quercetin. Pulegone, however, shows greater potential. Kaushik (Kaushik 2021) reported pulegone’s strong binding with Glypican-1 (GPC1), a protein linked to tissue repair (Abdelhady 2015; Ghatak et al. 2015; Melrose 2016). This compound also fulfilled Lipinski's rules for drug-likeness (Ivanović et al. 2020), reinforcing its potential as a dermatoprotective agent.

In contrast, in 2019, Maurya and collaborators studied the interaction of pulegone with pro-inflammatory cytokine receptors, specifically TNF-α and IL-6, comparing these results with those of dexamethasone, a reference steroidal anti-inflammatory drug (Maurya et al. 2019). Indeed, pro-inflammatory proteins play a key role in biological processes related to antidiabetic and dermatoprotective activities. The results revealed that pulegone exhibits a strong BA with pro-inflammatory cytokines, comparable to that of dexamethasone, as indicated by the low docking energy (Maurya et al. 2019). This suggests that pulegone could be a promising candidate for the development of effective anti-inflammatory treatments and, consequently, improve diabetes management and skin health.

To better understand menthone’s antibacterial mechanisms, Mirzaie et al. (Mirzaie et al. 2012) found that it fits well into the active site of nitro-reductase (NR), an enzyme involved in bacterial defense by reducing nitro-containing antibiotics. Menthone binds mainly through hydrophobic interactions with phenylalanine (Phe-124). A more recent study on MRSA used network analysis and molecular docking to explore antibacterial activity (Zhao et al. 2023b). It identified strong interactions (r > 0.98) between specific lipid species involved in MRSA inhibition and constructed a network of 85 predicted molecular targets. Docking results showed menthone effectively binds to key targets with significant negative binding energies, indicating that lipid-target interactions contribute to its antibacterial effect.

In summary, the findings of this investigation underscore the promising potential of pulegone and menthone as antidiabetic agents. Regarding their dermoprotective properties, both compounds showed significant interactions with key enzymes involved in skin health. These results highlight the need for further research to deepen our understanding of the diverse mechanisms of action of pulegone and menthone. Further studies should explore their full therapeutic potential in various contexts, including their roles in anti-inflammatory treatments and their impact on diabetes management and skin health. A comprehensive and multidisciplinary approach will elucidate the broader applications of these compounds in therapeutic contexts, opening new perspectives for the development of innovative treatments.

## Conclusions and perspectives

The in-depth study on MPEO has yielded particularly promising results, shedding light on several essential pharmacological aspects. Among the significant findings, menthone and pulegone have emerged as the main components of MPEO, holding substantial pharmacological potential. The conducted trials have highlighted their remarkable efficacy in the antibacterial domain, with impressive results against *S. aureus* and *L. monocytogenes*, suggesting their capacity to effectively inhibit bacterial growth. Furthermore, menthone has shown promising antidiabetic properties, marked by significant inhibition of α-amylase and α-glucosidase, thus emphasizing its potential role in glycemic regulation. In terms of skin protection, MPEO has exhibited strong dermatoprotective potential, with a notable capacity to inhibit key enzymes such as tyrosinase and elastase, suggesting its potential application in skincare products. Additionally, thorough in silico analysis has confirmed the strong affinity of pulegone and menthone towards enzymes involved in glucose regulation, further reinforcing the promising in vitro results. Overall, these conclusions open exciting new research perspectives on the potential use of MPEO in the development of medicines and skincare products. The growing importance of MPEO in the fields of natural medicine and cosmetics is underscored by its diverse bioactivities. Future research should focus on elucidating the mechanisms underlying these effects, optimizing extraction methods, and evaluating the clinical efficacy and safety of MPEO and its components. Moreover, the bioactive compounds that were evaluated separately were found to be potential candidates for pharmaceutical uses, particularly those ones that have exhibited strong antibacterial and anti-diabetic properties. Similar to the previous point, chemicals that have shown powerful dermatoprotective characteristics have great potential for use in cosmetic applications. These application possibilities have been discussed in the portion of the text that is devoted to conclusions and future prospects. This could lead to the development of innovative therapeutic and cosmetic applications, harnessing the full potential of MPEO as a natural remedy.

## Data Availability

All data were used a Stnd cited in the manuscript.
